# The Diagnostics of Disease Relapse Within Five-Year Follow-Up and the Role of Androgen Receptor Expression in Patients with Early Triple-Negative Breast Cancer

**DOI:** 10.3390/diagnostics15060692

**Published:** 2025-03-11

**Authors:** Igor Spurnić, Snežana Šušnjar, Irena Jovanić, Nataša Medić-Miljić, Zorka Milovanović, Marina Popović Krneta, Zoran Bukumirić, Dušica Gavrilović, Saša Rajšić, Ivan Marković

**Affiliations:** 1 Surgical Oncology Clinic, Institute for Oncology and Radiology of Serbia, 11000 Belgrade, Serbia; 2 Faculty of Medicine, University of Belgrade, 11000 Belgrade, Serbia; zoran.bukumiric@med.bg.ac.rs; 3 Clinic for Medical Oncology, Institute for Oncology and Radiology of Serbia, 11000 Belgrade, Serbia; 4 Department of Pathology, Institute of Oncology and Radiology of Serbia, 11000 Belgrade, Serbia; jovanicirena@gmail.com (I.J.); natasamm76@gmail.com (N.M.-M.); zmilovanovic@ncrc.ac.rs (Z.M.); 5 Department of Nuclear Medicine, Institute for Oncology and Radiology of Serbia, 11000 Belgrade, Serbia; marina.popovic1989@gmail.com; 6 Institute of Medical Statistics and Informatics, 11000 Belgrade, Serbia; 7 Data Center, Institute for Oncology and Radiology of Serbia, 11000 Belgrade, Serbia; duca.gavrilovic@gmail.com; 8 Department of Anesthesiology and Intensive Care Medicine, Medical University Innsbruck, 6020 Innsbruck, Austria

**Keywords:** triple-negative breast cancer, androgen receptor, overall survival, disease-free interval, disease-free survival

## Abstract

**Background/Objectives**: Triple-negative breast cancer (TNBC) is characterized by the absence of the expression of estrogen receptors, progesterone receptors, and human epidermal growth factor receptor 2. As there are no specific targeted therapies, TNBC patients often face an aggressive clinical course. The expression of the androgen receptor (AR) has been found in up to 30% of TNBC cases, but the association between the AR status and survival rates in TNBC remains controversial. The aim of this study was to explore the association of AR expression with the disease outcome in patients with early TNBC within a 5-year follow-up. **Methods**: AR expression was determined by immunohistochemistry in a cohort of 124 early-TNBC patients treated at the Institute for Oncology and Radiology of Serbia. The cut-off value used for the positive AR status was >10% tumor cells. The association of the AR status with clinicopathological factors (age, stage, tumor diameter, lymph node invasion, metastatic spread, Ki-67 score, EGFR score, and cytokeratin 5/6 score) and the disease outcome (disease-free survival—DFS—and overall survival—OS) was investigated. **Results**: Our analysis showed that the AR-positive status was associated with a significantly lower Ki-67 score compared to the AR-negative samples. A univariate analysis indicated that the age, tumor size, nodal status, and EGFR score significantly influenced both 5-year DFS and OS. Multivariate Cox analysis suggested that a smaller tumor size, lower nodal status, and AR expression were independent predictors of longer survival rates in TNBC patients. **Conclusions**: The results of this study suggest that the positive AR status may be a favorable prognostic factor in TNBC patients within the first five years after surgery.

## 1. Introduction

Triple-negative breast cancer (TNBC) is characterized by the absence of estrogen receptors (ER) and progesterone receptors (PR) expression, and the absence of the overexpression of the human epidermal growth factor receptor 2 (HER-2). TNBC represents approximately 15% of all breast cancer cases, is more common in younger, premenopausal women, and is associated with a shorter overall survival (OS) rate compared to other breast cancer subtypes [1]. The disease typically exhibits an early recurrence peak, around three years post-diagnosis, in the form of distant metastases, and has the highest mortality within the first five years after diagnosis [2]. Due to limited therapeutic options for TNBC, there is an urgent need for new therapeutic targets and prognostic biomarkers that could improve patient outcomes.

TNBC exhibits significant heterogeneity, with four distinct subtypes that influence prognosis and treatment strategies [3]. The luminal androgen receptor (LAR) subtype is characterized by increased levels of AR protein and mRNA, the upregulation of AR-regulated and luminal genes, a higher prevalence of *PIK3CA* mutations, the deletion of *PD-L1* (*CD274*) and *PD-L2* (*PDCD1lG2*) genes, and a low genomic scar signature score [3,4]. Although AR is overexpressed in 70–90% of all breast cancers, including up to 30% of TNBC [5], its contribution to TNBC aggressiveness and chemotherapy sensitivity remains controversial [1]. This is in line with its versatile functions within the tumor cell. As a nuclear hormone receptor, AR activated by ligand (dihydrotestosterone) binding is a transcription factor that regulates the expression of target genes predominantly involved in the control of cell proliferation, motility, and apoptosis. Preclinical studies indicate that AR upregulates the expression of the tumor suppressor gene *PTEN*, thus mediating androgen-induced growth inhibition and apoptosis in breast cancer cells [6]. Conversely, AR can also promote tumor progression by inducing the expression of metalloproteinases, thus facilitating extracellular matrix degradation, and enhancing tumor cells’ migration and invasiveness [7]. In prostate cancer, it was shown that AR can promote DNA repair through homologous recombination as its inhibition can mimic the loss-of-function of the breast cancer type 1 susceptibility protein (BRCA1) and confer cancer sensitivity to poly(ADP-ribose) polymerase 1 (PARP1) inhibitors. This suggests that PARP1 inhibitors, used to treat BRCA1-negative TNBCs, may be applied to threaten the LAR subtype of TNBC [8].

AR inhibition in TNBC represents a promising target since preclinical studies showed that antiandrogens induce an antiproliferative effect and derive a clinical benefit [9,10]. Currently there are several phase I/II studies investigating the efficacy and safety of various antiandrogens, and their combination with other targeted therapies such as CDK4/6 inhibitors, PI3K inhibitors, or immunotherapy [9]. Besides chemotherapy, the only novel drugs recommended by current guidelines for the treatment of early-stage or metastatic TNBC are immune checkpoint inhibitors or antibody–drug conjugates and PARP inhibitors for patients with germline BRCA1 and 2 deleterious mutations [11].

The prognostic significance of AR expression is still a matter of clinical research, since clinical findings are inconsistent. A meta-analysis published by Wang C. et al. [12] concluded that higher AR expression was associated with a better disease outcome and lower risk for recurrence. However, studies published afterwards conferred controversial results, some showing a better outcome [13,14,15,16,17], some a worse prognosis [18,19], while most did not show AR expression to be of any prognostic significance [20,21,22,23,24,25]. In line with these results, the most recently published systematic review and meta-analysis regarding the prognostic role of AR expression, which included 27 studies with 4914 patients with TNBC, did not show the association of AR expression with prognosis [26]. There are several factors potentially influencing differences in the AR prognostic significance between studies, such as a different cutoff for an AR-positive status, different methods for AR detection (e.g., different antibodies), or different statistical tests used [27]. Furthermore, the definition of TNBC using ER and PR protein expression (<1% versus <10%) may influence results, since it has been shown that AR inhibits cell proliferation in ER-expressing cancers through an ER signaling blockade [28]. While AR expression may influence tumor characteristics, its direct impact on prognosis is still under investigation and this study contributes valuable insights into the role of AR in TNBC prognosis within our cohort.

## 2. Materials and Methods

### 2.1. Patients

This study included 124 patients diagnosed with early-stage and locally advanced TNBC, who underwent surgical resection at the Institute of Oncology and Radiology of Serbia from 2009 to 2014. The patients were treated with either mastectomy or breast-conserving surgery, followed by postoperative radiation therapy. Adjuvant therapy was administered in accordance with the established guidelines for the diagnosis and treatment of breast cancer [29,30,31,32]. After the completion of the initial treatment for early TNBC, all patients were followed up regularly with clinical exams and annual mammography. This study includes disease outcome follow-ups until the end of 2022.

This study was approved by the Medical Ethics Committee of the School of Medicine, University of Belgrade (No 1322/VII-13, date 8 July 2021).

### 2.2. Histopathological Characteristics of the Tumor

All tumor samples, obtained after initial surgical treatment, were formalin-fixed and paraffin-embedded (FFPE). Primary tumor histopathology was determined using hematoxylin and eosin (H&E)-stained serial sections. Histological type, histological grade, tumor size, and axillary lymph node status were documented. Tumors were classified according to the WHO histological classification [33] and graded using the Nottingham grading system [34]. Pathological staging was performed according to the pTNM classification [35].

### 2.3. Immunohistochemical Analyses

#### 2.3.1. Automated Immunohistochemical Method

Estrogen receptor (ER), progesterone receptor (PR), HER2, Ki-67, AR, and CK5/6 immunohistochemical expression status were analyzed on 4 μm sections of FFPE tissues using the following antibodies: clone EP1 (Dako (Agilent), Santa Clara, CA 95051, USA), clone PgR636 (Dako), clone 4B5, (Ventana), clone 30-9 (Ventana, Tucson, AZ 85755, USA), clone SP107 (Cell Marque, Rocklin, CA 95677, USA), and clone D5/16B4 (Dako), respectively. The Autostainer Link 48 (Dako) was used for immunohistochemical staining of ER, PR, and CK5/6, and the BenchMark-GX Autostainer (Ventana) was used for HER2, Ki-67, and AR. Detection and visualization were performed using En Vision FLEX, High pH (Dako) for ER, PR, and CK5/6, and Ultra View Universal DAB Detection Kit (Ventana) for HER2, Ki-67, and AR.

#### 2.3.2. Manual Immunohistochemical Method

The manual immunohistochemistry technique was employed to detect EGFR expression. Tissue sections were first deparaffinized, then rehydrated through graded alcohol solutions, followed by distilled water. Next, they were incubated in 3% hydrogen peroxide for 10 min. Slides were placed in a pH 8 buffer and heated in a microwave at 95 °C for 29 min for antigen retrieval. Subsequently, tissue slides were left to cool at room temperature for 10 min and rinsed with phosphate-buffered saline. This was followed by the application of the primary monoclonal antibody (EGFR.113, Novocastra, 1:14). The UltraVision Quanto Detection System HRP + DAB Quanto (Epredia, Kalamazoo, MI 49008, USA) which was used for visualization.

### 2.4. Evaluation of Immunohistochemical Staining

Immunodetection of ER, PR, CK5/6, and EGFR were assessed using the Allred scoring system. This system combines the score for the percentage of stained malignant cell nuclei (0 for no immunoreactivity, 1 for <1% stained nuclei, 2 for 1–10%, 3 for 11–33%, 4 for 34–66%, and 5 for 67–100%) with the staining intensity score (0 for no immunoreactivity, 1 for weak intensity, 2 for moderate intensity, and 3 for strong intensity) [36,37]. ER and PR were considered negative if the score was <3 [38]. Score ≥ 4 was considered as positive for CK5/6 and EGFR [37].

HER-2 immunoreactivity was evaluated by assessing membrane staining, specifically the intensity and continuity, in tumor cells. A score of 0 indicated no staining or weak, incomplete staining in ≤10% of invasive tumor cells; a score of 1+ indicated weak, incomplete staining in >10% of invasive tumor cells; a score of 2+ indicated weak-to-moderate, complete staining in >10% or strong, complete, and circumferential staining in ≤10%; and a score of 3+ indicated strong, complete, circumferential staining in >10% of invasive tumor cells. HER-2 2+ tumors were further tested for HER2 amplification using CISH, Dual-CISH, or Dual-SISH methods [39,40].

The scoring system for Ki-67 was defined as the percentage of stained nuclei of malignant cells of any intensity. The cutoff value for the low Ki-67 proliferative index was ≤14% [41].

**Figure 1:**
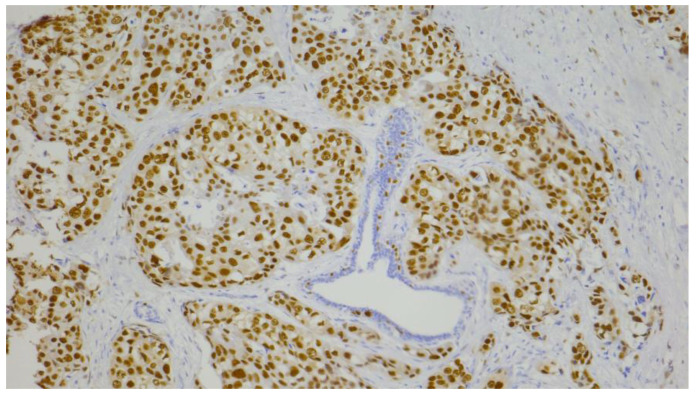
Immunohistochemical staining of AR (positive result): nuclear expression in 95% of tumor cells (100×).

**Figure 2:**
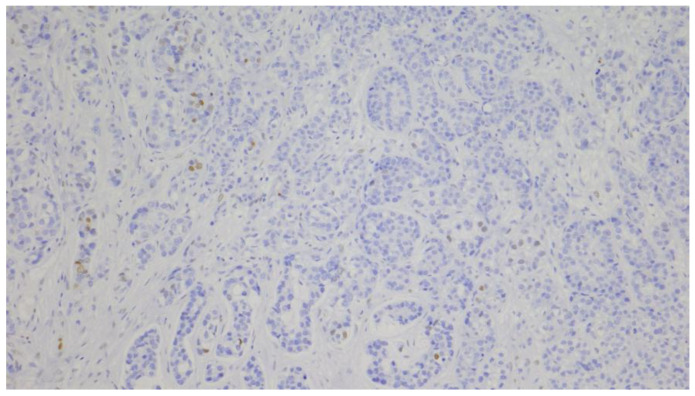
Immunohistochemical staining of AR (negative result): nuclear expression in <10% of tumor cells (100×).

The AR expression status was determined using a quantitative method that measured the percentage of malignant cells showing nuclear expression at any intensity level. IHC scoring was assessed by two independent pathologists. In cases of disagreement, a third pathologist reviewed the slides to reach a consensus. The cut-off value used for positive expression was >10% [42]. Representative images of positive (Figure 1) and negative AR expression (Figure 2) are shown.

### 2.5. Statistical Analysis

Statistical analysis was performed using IBM SPSS Statistics 24 (IBM Corporation, Armonk, NY, USA) and R-4.3.3 software (The R Foundation for Statistical Computing, Vienna, Austria). Depending on the type of variables and the normality of distribution, data description is presented as *n* (%), mean ± standard deviation, or median (min–max). The methods used for testing statistical hypotheses included: *t*-test, Mann–Whitney test, chi-square test, and Fisher’s exact test.

Time-to-event outcomes were defined as follows: (a) DFS as time from surgery to invasive ipsilateral breast cancer relapse, regional relapse, contralateral breast cancer, distant metastases, or death w/o disease relapse, and (b) OS as time from surgery to death due to any cause. For these analyses, the Kaplan–Meier method was used. Considering higher risk of TNBC relapse in the first several years from the diagnosis, univariate and multivariate analyses were limited to the first 5 years after surgery.

To identify independent predictors of outcomes of interest (relapse and mortality), Cox proportional hazards regression model with a 95% confidence interval was applied. The variables included in the multivariate Cox regression models were those that were statistically significant at the 0.1 significance level in the univariate models and selected through LASSO (Least Absolute Shrinkage and Selection Operator) regularization. All *p*-values less than 0.05 were considered significant.

## 3. Results

### 3.1. Patients, Disease, and Therapy Characteristics of the TNBC Cohort

The cohort included 124 TNBC patients, with a median age of 58.5 (range 30–88) years. Most patients had tumors measuring between 2 and 5 cm. A regional lymph node metastasis (LNM) was histologically confirmed in 36.6% patients. Specifically, 36 patients had one to three LNM, while 9 patients had more than four. The evaluated tumors displayed a high degree of malignancy, with grade III tumors comprising over half of the cases (53.2%) and 79% of tumors exhibiting a Ki-67 index greater than 30%. The immunoreactivity results for CK5/6 and EGFR, quantified using the scoring systems, showed that 62.1% of patients had a negative CK5/6 score and 66.1% had a negative EGFR score. Tumors exhibiting a basal-like status were defined using the combined expression of EGFR and CK5/6. The characteristics of the patients and tumors are presented in Table 1.

**Figure 3:**
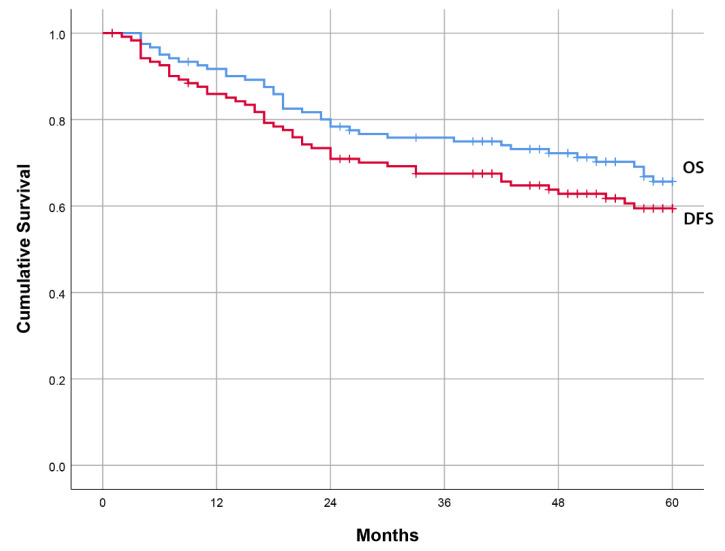
Kaplan–Meier Curves for 5-Year Overall Survival (OS) and Disease-Free Survival (DFS).

The therapy characteristics and disease outcomes are also shown in Table 1. Among the evaluated patients, 43.5% underwent breast-conserving surgery, while 56.5% received a mastectomy. Additionally, 73.4% received adjuvant chemotherapy and 65.3% were treated with adjuvant radiotherapy. During the follow-up period of 60 months, 29.8% of patients experienced a disease relapse, while 38.7% of patients died. The estimated mean values for DFS and OS in the 5-year follow-up period were 44.3 months and 48.3 months, respectively (Figure 3).

### 3.2. Correlation of Clinicopathological Features, Treatment Characteristics, and Androgen Receptor Status

The clinicopathological and treatment characteristics with respect to the AR status are reported in Table 2. AR expression was significantly inversely correlated with Ki-67, with the negative AR status associated with a higher Ki-67 score (*p* < 0.001). No significant differences were observed between the AR status and any other evaluated factor.

### 3.3. Univariate Analyses of Prognostic Factors for 5-Year Disease-Free Survival and Overall Survival

Univariate Cox regression was used to evaluate the impact of various clinicopathological and treatment characteristics on the 5-year DFS and OS of patients, and the results are presented in Table 3. The analysis revealed that the age, tumor size, nodal status, and EGFR score significantly influenced both the 5-year DFS and OS (*p* < 0,05). Specifically, these characteristics were associated with poorer outcomes. Elevated levels of CK5/6 were associated with worse 5-year OS, while no significant association was observed with the 5-year DFS. Patients who received adjuvant chemotherapy demonstrated improved survival rates, indicating its beneficial effect on patient prognosis. Similarly, adjuvant radiotherapy was associated with improved 5-year OS, though its impact on DFS was not statistically significant.

**Figure 4:**
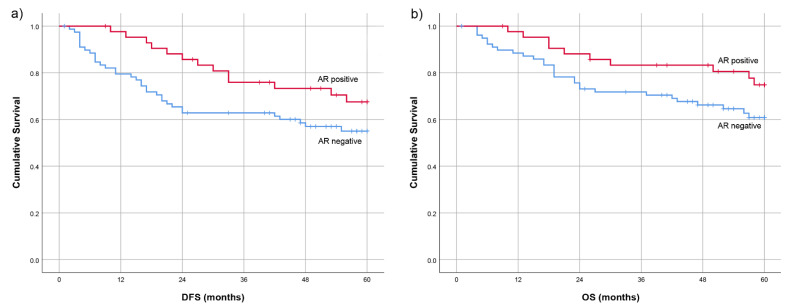
Kaplan–Meier curves for impact of negative and positive AR expression on the 5-year (**a**) Disease-Free Survival (DFS) and (**b**) Overall Survival (OS).

To further illustrate the impact of the AR status, Kaplan–Meier curves were generated for both the 5-year DFS and OS. The Log-Rank test did not reveal statistically significant differences between AR-positive and AR-negative groups (DFS: *p* = 0.084; OS: *p* = 0.092). These findings are presented in Figure 4.

### 3.4. Multivariate Cox Regression Analysis of Prognostic Factors for 5-Year Disease-Free Survival and Overall Survival

**Figure 5:**
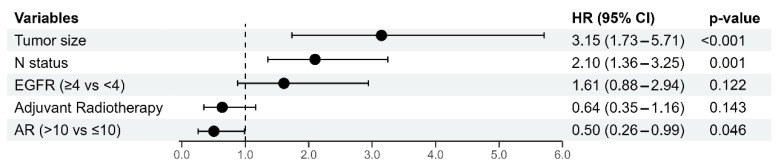
Multivariate Cox Regression Analysis of Predictors for 5-year Disease-Free Survival. Abbreviations: N: nodal status; EGFR: Epidermal Growth Factor Receptor; AR: Androgen Receptor; HR = hazard ratio.

In multivariate Cox regression models, the tumor size, the N status, and the AR status were identified as independent predictors of DFS. A larger tumor size (B = 1.147; *p* < 0.001) and a higher number of positive lymph nodes (B = 0.742, *p* = 0.001) were both associated with an increased recurrence hazard ratio, highlighting their prognostic relevance for shorter DFS. Conversely, a positive AR status correlated with a reduced recurrence hazard ratio (B = −0.682, *p* = 0.046), suggesting favorable prognostic significance for DFS. The results are presented in Figure 5.

**Figure 6:**
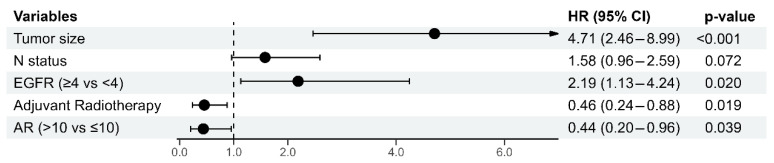
Multivariate Cox Regression Analysis of Predictors for 5-year Overall Survival. Abbreviations: N: nodal status; EGFR: Epidermal Growth Factor Receptor; AR: Androgen Receptor; HR = hazard ratio.

The multivariate analysis for the 5-year OS identified the tumor size (B = 1.549, *p* < 0.001) and EGFR status (B = 0.783, *p* = 0.020) as independent predictors of mortality risk, with larger tumors and the EGFR-positive score increasing the hazard of death. In contrast, adjuvant radiotherapy (B = −0.783, *p* = 0.019) and the positive AR status (B = −0.822, *p* = 0.039) were both significantly associated with a better 5-year OS. The results are presented in Figure 6.

## 4. Discussion

The results of this study indicate that the positive AR status in TNBC was associated with a lower recurrence and death hazard ratio during the 5-year follow-up, with a strong inverse correlation between AR expression and cancer cell proliferation (Ki-67 index). This study identifies the tumor size, nodal status, and the EGFR score as independent predictors for poorer DFS and OS, while adjuvant chemotherapy and radiotherapy were associated with improved survival rates. With respect to the clinicopathological factors and treatments used, the AR-positive and AR-negative subgroups were well balanced. Although time-to-event curves were separated early between the AR-positive and AR-negative subgroups, there were no statistically significant differences in DFS and OS between these two subgroups. The reasonable explanation for this result lies in the small number of patients within the analyzed cohort and a small number of events (disease relapse or death).

The AR status is not routinely assessed in breast tumors. Currently, for an immunohistochemical analysis of the AR status, various antibodies with a broad range of cutoff values are used [15,43]. This variability complicates the interpretation of the AR’s role in breast cancer when relying solely on the immunohistochemistry-based expression data and could underly conflicting reports on the prognostic value of AR expression in TNBC. A previous study reported that a higher percentage of AR-positive breast cancer cells was associated with a better OS [44]. We chose to set the AR-positivity cutoff to 10% tumor cells because this was the most frequently observed prevalence of AR-positivity in the reported studies [45]. The available literature on the association of the AR-positive status and disease outcome in breast cancer patients is inconclusive. One meta-analysis showed that AR expression correlated with worse OS, without any significant effect on DFS [46]. Another study found AR expression in TNBC to be associated with a longer DFS and OS [12]. A recently reported systematic review and meta-analysis found no impact of AR expression on TNBC disease outcome [26]. The results of one meta-analysis [28] showed that the AR-positive status was more frequently observed among ER-positive compared to ER-negative early breast cancers. While a crosstalk between ERs and ARs in the luminal ER-positive breast cancer leads to ER signaling inhibition, thus improving disease prognosis, ARs in TNBCs enhance proliferative signaling by binding to estrogen response elements on DNA [28,47]. AR expression in TNBC was detected not only in LAR, but also in non-LAR TNBC subtypes [9]. This may contribute to inconsistent results regarding a more favorable prognosis of LAR tumors.

In addition to the importance of AR for the TNBC outcome, our study also identifies EGFR expression as an independent prognostic factor for decreased DFS and OS. This is in line with other studies, suggesting that EGFR overexpression in breast cancer is associated with a larger tumor size, reduced tumor cell differentiation, and poor clinical outcomes [48,49]. Although EGFR overexpression has been reported in all types of breast cancer, it is more frequent in TNBC, where it is found in up to 70% of patients [50,51,52]. A combined AR and EGFR status makes a distinction between AR-positive and EGFR-negative vs. AR-negative and EGFR-positive subgroups, with the latter subgroup having a poorer prognosis and being more likely to benefit from chemotherapy [45].

Other tumor characteristics such as a lower grade, lower mitotic score, less frequent metastasis, and tumor recurrence [4,53,54,55,56], correlated well with AR positivity. These results align with this study’s findings, where AR was identified as an independent predictor of longer DFS and OS at 5 years. Similar to the earlier studies, our results indicate that both a larger tumor size and positive nodal status were independent poor prognostic factors for DFS and OS during the 5-year follow-up.

The AR status was found to be inversely proportional to the mitotic score in TNBC in our cohort of patients. This negative correlation between AR and Ki-67 has been reported in several studies [4,42,57,58]. This finding is in line with the results of the recently published meta-analysis showing that the response to neoadjuvant chemotherapy including taxanes and anthracyclines is lower in the LAR subtype of TNBC [59]. As far as the antineoplastic postoperative therapy is concerned, the univariant analysis showed that the adjuvant chemotherapy significantly improved both DFS and OS, which is expected. However, its role as an independent prognostic factor was not confirmed. About half of our patients received adjuvant anthracycline-containing chemotherapy and less than 25% received a combination of anthracyclines followed by taxanes. Due to the small number of patients in our cohort, the potential AR status-dependent responsiveness to chemotherapy could not be analyzed. The postoperative radiotherapy was found to be a predictor of prolonged DFS and OS in the entire investigated patient population. In contrast, a previously published study reported that the AR-positive breast cancers are resistant to radiotherapy and that the local recurrence is more frequent in AR-positive breast cancer patients who underwent postoperative irradiation [45].

There is a complex interplay between AR signaling and cell proliferation. Namely, when activated with a low dose of androgens, AR is predominantly in the form of a monomer and, as such, it activates mTOR signaling to drive proliferation. In contrast, high-dose androgens facilitate the formation of AR dimers/oligomers to suppress c-MYC expression, inhibit proliferation, and drive a transcriptional program associated with a differentiated phenotype of AR-positive prostate cancer cell lines [60]. When it comes to TNBC, the question about the quantity of androgens in women arises. Therefore, the observed better prognostic prospects in the case of AR positivity may be the consequence of an inherently higher testosterone concentration in these women. The contradictory response to different concentrations of AR ligands may help explain the diversity in treatment outcomes observed in both animal models and clinical trials. However, numerous preclinical studies point to the benefits of AR targeting for TNBC treatment. For example, the combination of an antiandrogen drug Bicalutamide and radiotherapy significantly attenuated tumor growth in the AR-positive compared to the AR-negative animal model [61]. It was also shown that the AR blockade with antiandrogens such as enzalutamide may inhibit DNA repair, rendering tumor cells more sensitive to radiotherapy-induced damage [45].

The combination treatment with the cell cycle CDK4/6 inhibitor Abemaciclib and the androgen receptor inhibitor Seviteronel showed a synergistic effect in an AR-positive TNBC model [62]. The most recent phase II clinical trial introduces the AR inhibitor Enzalutamide as an oral drug for LAR, with no unexpected side effects and with significant efficacy in combination with Paclitaxel [63]. These findings underscore the significance of AR expression in TNBC for the development of enhanced therapeutic options. In this context, the current study in our patient cohort provides valuable insights into the AR distribution in TNBC and its association with survival rates, adding to the general knowledge about TNBC physiology.

AR-positive TNBC has not been recognized as a distinct entity because its prognostic and predictive significance has not been well established. While limited data do exist about antineoplastic AR-targeted antiandrogen therapy such as Bicalutamide, Enzalutamide, and Abiraterone, alone or in combination with chemotherapy, PIK3CA inhibitors (Alpelisib), and CDK4/6 inhibitors (Palbociclib and Ribociclib) [64], until the results from the phase 3 clinical studies become available, the AR-targeted therapy should only be considered as an exploratory approach. Although targeting AR in TNBC carries several challenges such as a lack of standardized testing, the heterogeneity of TNBC, limited clinical data, and adverse effects, addressing these challenges is essential for the successful integration of AR-targeted therapies into future clinical practice, aiming to improve outcomes for patients with LAR.

## Figures and Tables

**Table 1:** Summary description of the original dataset.

Charasteristics	Median (Range)
Age (years)	58.5 (30–88)
Tumor size (cm) *	*n* (%)
≤2	37 (29.8)
2–5	75 (60.5)
>5	11 (8.9)
Unknown	1 (0.8)
N (number of LNM) **	*n* (%)
0	78 (62.9)
1–3	36 (29.0)
4+	9 (7.3)
Unknown	1 (0.8)
Tumor grade	*n* (%)
II	58 (48.6)
III	66 (53.2)
Ki-67 % score	*n* (%)
≤14	12 (9.7)
15–30	14 (11.3)
≥30	98 (79.0)
Androgen receptor (AR)	*n* (%)
negative (≤10%)	81 (65.3)
positive (>10%)	43 (34.7)
Cytokeratin (CK) 5/6 score	*n* (%)
negative (<4)	77 (62.1)
positive (≥4)	47 (37.9)
EGFR score	*n* (%)
negative (<4)	82 (66.1)
positive (≥4)	42 (33.9)
Tumor type	*n* (%)
non-basal	57 (46.0)
basal-like	67 (54.0)
Type of surgery	*n* (%)
breast-conserving surgery	54 (43.5)
mastectomy	70 (56.5)
Adjuvant chemotherapy	*n* (%)
no	33 (26.6)
yes	91 (73.4)
Adjuvant radiotherapy	*n* (%)
no	43 (34.7)
yes	81 (65.3)
Disease relapse	*n* (%)
no	87 (70.2)
yes	37 (29.8)
Patients’ outcome	*n* (%)
Alive	76 (61.3)
Died	48 (38.7)
Total	124 (100%)
5-year FUP	Mean value (95%CI)
DFS (months)	44.3 (40.5–48.2)
OS (months)	48.3 (44.9–51.7)

* For one patient unknown tumor size; ** for one patient unknown number of positive lymph nodes; Abbreviations: N: nodal status; LNM: lymph node metastasis; EGFR: Epidermal Growth Factor Receptor; FUP: follow-up period; DFS: Disease-Free Survival; OS: Overall Survival.

**Table 2:** Clinicopathological and treatment characteristics with respect to the AR status.

Charasteristics	AR-Negative	AR-Positive	*p*-Value
	(*n* = 81)	(*n* = 43)	
Age (years; mean ± SD)	57.7 ± 11.9	59.9 ± 11.3	0.318
Tumor size (cm)	*n* (%)	*n* (%)	0.051
≤2	21 (25.9)	16 (37.2)	
2–5	53 (65.4)	22 (51.2)	
>5	7 (8.6)	4 (9.3)	
Unknown	0	1 (2.3)	
N status (number of LNM)	N (%)	N (%)	0.858
0	51 (63.0)	27 (62.8)	
1–3	23 (28.4)	13 (30.2)	
4+	6 (7.4)	3 (7.0)	
Unknown	1 (1.2)	0	
Tumor grade	*n* (%)	*n* (%)	0.065
II	33 (40.7)	25 (58.1)	
III	48 (59.3)	18 (41.9)	
Ki-67 % score	*n* (%)	*n* (%)	<0.001 *
≤14	1 (1.2)	11 (25.6)	
15–30	6 (7.4)	8 (18.6)	
>30	74 (91.4)	24 (55.8)	
Cytokeratin (CK) 5/6 score	*n* (%)	*n* (%)	0.371
negative (<4)	48 (59.3)	29 (67.4)	
positive (≥4)	33 (40.7)	14 (32.6)	
EGFR score	*n* (%)	*n* (%)	0.822
negative (<4)	53 (65.4)	29 (67.4)	
positive (≥4)	28 (34.6)	14 (32.6)	
Type of surgery	*n* (%)	*n* (%)	0.917
Breast-conserving surgery	35 (43.2)	19 (44.2)	
Mastectomy	46 (56.8)	24 (55.8)	
Adjuvant Chemotherapy	*n* (%)	*n* (%)	0.850
No	22 (22.7)	11 (25.6)	
Yes	59 (72.8)	32 (74.4)	
Adjuvant radiotherapy	*n* (%)	*n* (%)	0.972
No	28 (34.6)	15 (34.9)	
Yes	53 (65.4)	28 (65.1)	

Abbreviations: N: nodal status; LNM: lymph node metastasis; EGFR: Epidermal Growth Factor Receptor; SD: standard deviation. * Statistically significant (*p* < 0.05).

**Table 3:** Univariate Cox Analysis of Predictive Factors for 5-Year DFS and OS.

Charasteristics	5-Year DFS		5-Year OS	
	HR (95% CI)	*p* Value	HR (95% CI)	*p* Value
Age	1.04 (1.01–1.06)	0.010 *	1.04 (1.01–1.07)	0.007 *
Tumor size	3.18 (1.83–5.53)	<0.001 *	4.31 (2.37–7.85)	<0.001 *
Nodal status	2.23 (1.5–3.32)	<0.001 *	1.76 (1.13–2.73)	0.012 *
Tumor grade	0.61 (0.34–1.09)	0.092	0.64 (0.34–1.2)	0.165
Ki-67% score	0.76 (0.51–1.13)	0.171	0.95 (0.58–1.53)	0.822
AR (>10% vs. ≤10%)	0.57 (0.3–1.09)	0.089	0.55 (0.27–1.12)	0.099
CK 5/6 score (≥4 vs. <4)	1.33 (0.75–2.36)	0.333	2.07 (1.1–3.89)	0.024 *
EGFR score (≥4 vs. <4)	1.99 (1.12–3.54)	0.019 *	2.4 (1.28–4.5)	0.007 *
Type of surgery (yes vs. no)	0.82 (0.46–1.47)	0.501	0.62 (0.32–1.2)	0.154
Adjuvant chemotherapy (yes vs. no)	0.41 (0.23–0.74)	0.003 *	0.32 (0.17–0.6)	<0.001 *
Adjuvant radiotherapy (yes vs. no)	0.6 (0.33–1.06)	0.079	0.42 (0.22–0.78)	0.006 *

Abbreviations: AR: Androgen Receptor; CK: Cytokeratin; EGFR: Epidermal Growth Factor Receptor; HR: hazard ratio. *: Statistically significant (*p* < 0.05).

## Data Availability

Dataset available on request from the authors.
